# Management of Non-response and Loss of Response to Anti-tumor Necrosis Factor Therapy in Inflammatory Bowel Disease

**DOI:** 10.3389/fmed.2022.897936

**Published:** 2022-06-15

**Authors:** Jan Marsal, Manuel Barreiro-de Acosta, Irina Blumenstein, Maria Cappello, Thomas Bazin, Shaji Sebastian

**Affiliations:** ^1^Department of Gastroenterology, Skåne University Hospital, Lund/Malmö, Sweden; ^2^Department of Immunology, Lund University, Lund, Sweden; ^3^Gastroenterology Department, Hospital Clínico Universitario de Santiago de Compostela, Santiago de Compostela, Spain; ^4^Department of Internal Medicine 1, Gastroenterology, Hepatology and Clinical Nutrition, University Clinic Frankfurt, Frankfurt, Germany; ^5^Gastroenterology and Hepatology Section, Promise, University of Palermo, Palermo, Italy; ^6^Department of Gastroenterology, Université Paris Saclay/UVSQ, INSERM, Infection and Inflammation, UMR 1173, AP-HP, Hôpital Ambroise Paré, Boulogne Billancourt, France; ^7^Inflammatory Bowel Disease (IBD) Unit, Hull University Teaching Hospitals National Health Service (NHS) Trust, Hull, United Kingdom

**Keywords:** anti-TNF, loss of response, primary non-response, switch out of class, switch within class, therapeutic drug monitoring

## Abstract

Anti-tumor necrosis factor (anti-TNF) therapy has been successfully used as first-line biologic treatment for moderate-to-severe inflammatory bowel disease (IBD), in both “step-up” and “top-down” approaches, and has become a cornerstone of IBD management. However, in a proportion of patients the effectiveness of anti-TNF therapy is sub-optimal. Either patients do not achieve adequate initial response (primary non-response) or they lose response after initial success (loss of response). Therapeutic drug monitoring determines drug serum concentrations and the presence of anti-drug antibodies (ADAbs) and can help guide treatment optimization to improve patient outcomes. For patients with low drug concentrations who are ADAb-negative or display low levels of ADAbs, dose escalation is recommended. Should response remain unchanged following dose optimization the question whether to switch within class (anti-TNF) or out of class (different mechanism of action) arises. If ADAb levels are high and the patient has previously benefited from anti-TNF therapy, then switching within class is a viable option as ADAbs are molecule specific. Addition of an immunomodulator may lead to a decrease in ADAbs and a regaining of response in a proportion of patients. If a patient does not achieve a robust therapeutic response with an initial anti-TNF despite adequate drug levels, then switching out of class is appropriate. In conjunction with the guidance above, other factors including patient preference, age, comorbidities, disease phenotype, extra-intestinal manifestations, and treatment costs need to be factored into the treatment decision. In this review we discuss current evidence in this field and provide guidance on therapeutic decision-making in clinical situations.

## Introduction

Inflammatory bowel disease (IBD), broadly comprising Crohn’s disease (CD) and ulcerative colitis (UC), is a lifelong, debilitating condition necessitating a tailored and cost-effective approach to its management. Overarching therapeutic goals are to eliminate symptoms, avoid disease complications and optimize the patient’s quality of life (QoL) (1–5). By reaching certain therapeutic targets (the “treat-to-target” approach) it is believed that the chances of achieving these therapeutic goals are markedly improved. Recently, these therapeutic targets have evolved beyond symptomatic control to the normalization of objective markers of inflammation and endoscopic healing with the aim of modifying the disease course (5, 6).

**FIGURE 1:**
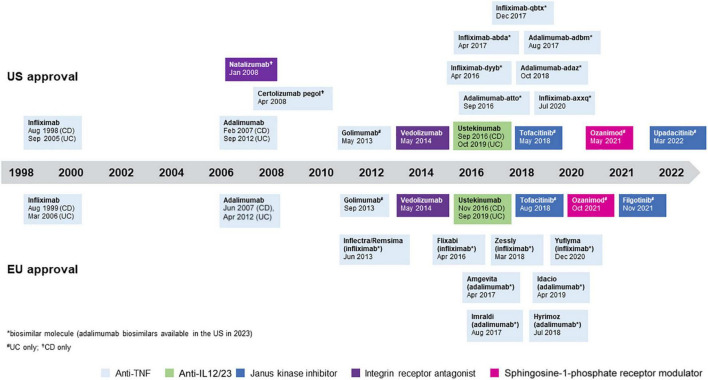
Approved treatments for inflammatory bowel disease (IBD). All treatments are approved for Crohn’s disease (CD) and ulcerative colitis (UC) unless otherwise specified.

There are two main strategies for the management of IBD. The “step up” approach is used for patients with mild-to-moderate disease without poor prognosis factors starting with conventional therapies (e.g., 5-ASA, azathioprine, methotrexate) before moving on to newer and more expensive biologic or small molecule treatments, all of which have specific side effects that need to be taken into account when choosing a therapy (7). An accelerated version of the step-up approach involves moving quickly upwards through traditional therapies, driven by predefined time-points for therapeutic evaluation with prespecified criteria for therapeutic targets. If these are not reached one goes quickly to the next level of therapy with the aim of avoiding prolonged periods of under-treatment, but still following the step-up approach (8). The “top down” approach has been proposed for patients with severe disease and a high risk for disease-related complications. It uses the most potent treatments available, including biologics and immunomodulators in combination, earlier in the disease course with the aim of inducing remission and maintaining corticosteroid-free remission (7, 9–11). Over the past two decades, anti-tumor necrosis factor (anti-TNF) therapy has been successfully used as first-line biologic treatment to treat moderate-to-severe IBD in both “step up” and “top down” approaches. In addition, the more recent introduction of vedolizumab, ustekinumab, and tofacitinib provide alternative first-line treatment options, although their use may be limited by regulatory and reimbursement constraints in some countries (1–4, 12–14). A summary of available treatments for IBD is shown in Figure 1.

The use of anti-TNFs has been shown to improve clinical symptoms, promote endoscopic healing, improve QoL and reduce hospitalizations and surgeries in patients with IBD (15, 16), benefits that can be increased by use early in the disease course, at least in CD (10, 15, 17). While anti-TNFs usually follow initial treatment in a step-up approach, in some patients with moderate-to-severe IBD and prognostic factors of unfavorable outcome (i.e., young age at diagnosis, perianal disease, penetrating disease in CD, and extensive disease) early anti-TNF and immunomodulator combination therapy may be beneficial (18–23).

Unfortunately, failure of anti-TNF therapy can occur and questions that naturally arise are whether regaining response with the current drug or drug class is possible and/or what the patient should be treated with next. This review explores the management of treatment options for IBD patients with a primary non-response (PNR) or loss of response (LOR) to anti-TNF therapy.

## Problem of Non-response and Loss of Response to Anti-tumor Necrosis Factors

### Primary Non-response

While there is no consensus definition of PNR it has been suggested to mean the failure to achieve a clinical response within 14 weeks of initiating treatment (1–4, 13). It has been reported that PNR to anti-TNFs occurs in 10–40% of patients with IBD (24–26). Primary non-response to anti-TNFs may be caused by a number of pharmacokinetic (drug concentrations) or pharmacodynamic (mechanistic) factors (6). Pharmacokinetic PNR is due to increased drug clearance, which may be immune mediated or non-immune mediated. It has also been shown that a proportion of administered anti-TNF is lost from the intestines of UC patients with active disease and that PNR is associated with the highest levels of anti-TNF observed in the feces (27). In contrast, pharmacodynamic PNR occurs when active disease persists despite therapeutic biologic drug levels, which implies that the binding of the drug to TNF is blocked or the presence of a non-inflammatory complication such as stenosis, abscess or a superimposed infection that has not been recognized; or that the underlying disease pathophysiology is primarily driven by inflammatory mediators other than TNF. Low albumin levels have been consistently associated with low infliximab levels and correlate with diminished clinical response, particularly in the setting of severe IBD such as in acute UC (28, 29).

### Loss of Response

Loss of response refers to those situations where patients respond to initial treatment with anti-TNFs but then subsequently and progressively lose this response. It has been reported that up to 50% of patients experience LOR over time and that the annual rate is ∼5–20% (30–33). The wide range of frequencies reported for LOR between studies can be explained by the differing definitions that have been used. These include those based on a worsening of symptoms, the need for dose escalation, an increased level of inflammation, stopping the drug, as well as differences depending on which anti-TNF agent is being studied (30). Loss of response to anti-TNFs may be related to low trough serum drug concentrations and/or the potential presence of anti-drug antibodies (ADAbs), which result in suboptimal drug concentrations (34) or a reduction in TNF-binding capacity (35). However, in some cases, other mechanisms such as the disease transitioning to other cytokine pathways are thought to cause LOR (12, 34).

### Clinical Assessment

In patients with a suspicion of PNR or LOR to anti-TNF therapy, guidelines suggest detailed assessment to determine the possible cause as this will guide therapeutic management options (1–4, 13). The first step is to determine whether the increase in symptoms is caused by a true increase in IBD activity or something else. Alternative causes for an increase in symptoms that should be ruled out include gastrointestinal infections, irritable bowel disease, bacterial overgrowth, and bile acid malabsorption (the latter being typically seen in patients with CD that have extensive ileal disease or have undergone ileal resection). The second step is to assess the level of disease-associated inflammatory activity present. A summary of various tools that can be used to assess inflammation is shown in Table 1.

**TABLE 1:** Available tools for assessing the level of disease-associated inflammatory activity.

Tool	Additional notes
Blood inflammatory markers (153, 154)	• Serum CRP and albumin can be used as parallel measures of disease severity/inflammation • CRP can be used as a prognostic marker for the effectiveness of therapy • ESR is a marker for inflammation but can be influenced by factors such as pregnancy, older age and anemia and is not widely used currently
Fecal biomarkers (1–4, 13, 155)	• Fecal calprotectin is a useful biomarker to assess the degree of mucosal inflammation • Fecal calprotectin is correlated with endoscopic inflammatory scores • Fecal calprotectin should be used in the management of patients with IBD
Endoscopy (156)	• “Gold standard” for assessing the response to treatment in patients with UC and CD
Histology (157)	• Endoscopic biopsies or resection specimens
Cross-sectional imaging (39, 158–162)	• MRI and computed tomography have a high sensitivity and specificity for assessing CD activity and can be used to monitor response to treatment • Bowel ultrasonography is increasingly being used in clinical practice ∙ Good correlation between bowel ultrasound findings and CD activity and location, as well as endoscopic remission ∙ Accurate method for assessing transmural healing, correlating well with time-consuming and costly MRI ∙ Convincing support for the use of ultrasonography as a monitoring tool for UC

*CD, Crohn’s disease; CRP, C-reactive protein; MRI, magnetic resonance imaging; UC, ulcerative colitis. ESR, erythrocyte sedimentation rate.*

## Options for the Therapeutic Management of Non-response to Anti-tumor Necrosis Factors

Given the still limited number of available therapies for IBD in 2021, early optimization of a patient’s current treatment and maintenance of clinical response/remission is important to avoid a rapid progression through therapeutic options. A key factor in this is assessment of adherence to treatment as this remains a critical factor in achieving and sustaining remission in IBD (36, 37). Patient-related factors that have been shown to be associated with poorer adherence to treatment include male sex, shorter IBD duration, and clinic non-attendances, Conversely, patients’ preferences have been shown to be important to consider to optimize adherence (36, 38).

**FIGURE 2:**
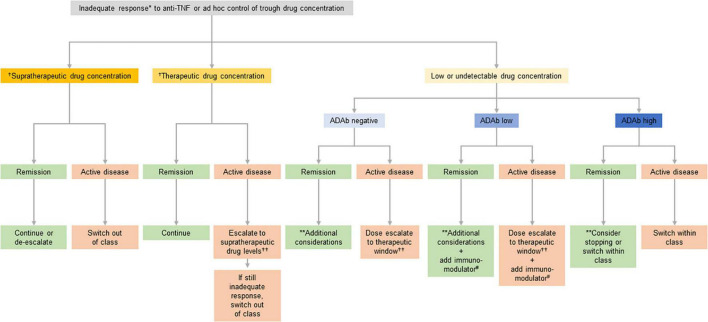
Suggested clinical therapeutic drug monitoring (TDM)-based algorithm for optimizing anti-tumor necrosis factor (anti-TNF) therapy. *If disease activity is defined by symptoms confirm inflammatory activity and/or rule out potential non-inflammatory causes. Potential non-inflammatory causes of increased symptoms include fibrotic stricture, gastrointestinal infection, irritable bowel syndrome, bacterial overgrowth, bile salt diarrhea, colorectal cancer, and andamyloidosis. **This situation may be interpreted either as: (A) the patient being in remission despite not having any relevant anti-TNF activity (low/undetectable drug concentration) and thus it may be stopped; or (B) the patient is in the first step toward a potential relapse according to the multi-step hypothesis suggesting that the first step toward a relapse is a decline in drug concentration, the second step an increase in subclinical inflammation, and the final step a clinical relapse, and thus the drug concentration should be brought back to the therapeutic window. Deciding on which of the two is most likely involves taking several aspects into account including the patient’s disease history, comorbidities, and concomitant medications. ^†^See Table 2 for suggested supratherapeutic and therapeutic drug concentrations. ††Both increase in dose (at standard doses) and increase in frequency are appropriate but maintaining the dose interval saves on nurse/infusion-related resources. #Immunomodulator defined as azathioprine or methotrexate.

Therapeutic drug monitoring (TDM) to determine drug trough serum concentrations and anti-drug antibodies (ADAbs) can help guide treatment optimization, improve outcomes of patients receiving anti-TNFs, and enhance cost efficiency (39–42). Treatment decisions where TDM may offer guidance include dose escalation, de-escalation or stopping, adding an immunomodulator, or switching to an alternative anti-TNF agent (switch within class) or a drug with a different mechanism of action (switch out of class). Such decisions can be made empirically but studies have shown that the use of TDM as a support for decision making is more cost-effective and provides better outcomes (43). An algorithm to guide the optimization of IBD therapy using TDM is shown in Figure 2 (42).

**TABLE 2:** Proposed target levels of anti-tumor necrosis factors (anti-TNFs) for clinical decision making based on published data and expert opinion.

Clinical time point	Infliximab	Adalimumab	Golimumab
After induction (week 14)	4–15 μg/mL (163)	N/D	N/D
During remission (therapeutic)	4–8 μg/mL (164–167)	5–10 μg/mL (163, 165–167)	1.4–4 μg/mL* (168–171)
To treat flare or before discontinuing due to loss of response (supratherapeutic)	>10 μg/mL (163)	>12 μg/mL (163)	N/D
For fistula healing	>12 μg/mL (172)	>14 μg/mL	N/A

N/A, not applicable; N/D, no consistent data; TDM, therapeutic dose monitoring; *Assay dependent.

TDM can be either reactive (occurs in response to treatment failure to guide therapy) or proactive [occurs at prescheduled time-points irrespective of disease activity to prevent LOR (39, 43)]. As this review discusses management options following failure of first-line anti-TNF, TDM here refers primarily to the reactive version.

### Optimizing Current Therapy in Primary Non-responders

TDM is recommended for patients suspected of experiencing PNR to anti-TNFs (41, 44). However, the results of TDM need to be reviewed alongside other factors to ensure that the patient is having a true PNR and that drug levels are not low due to other causes, including poor adherence. For primary non-responders to anti-TNF therapy with low drug concentrations and who are ADAb negative or low ADAb positive (as defined by the method used), dose escalation is recommended in an attempt to optimize symptom and inflammation management (Figure 2; 1–4, 12, 13).

### Optimizing Current Therapy Following Loss of Response

TDM is also recommended for patients suspected of having LOR to anti-TNFs (41, 44), as treatment optimization can be guided by TDM in a similar way as in the event of PNR (Figure 2). Dose escalation may reduce or even reverse the loss of therapeutic response to anti-TNF therapy (37, 45–47). Billioud et al. (45) reported that while one fifth of patients with CD experience a LOR after initiation of adalimumab therapy, dose escalation resulted in response recovery in the majority of patients. Similarly, adalimumab dose escalation enabled recovery of response in nearly half of patients with UC that had experienced LOR (47). In patients with CD with LOR to a standard infliximab dose, shortening the dosing interval from 8 to 6 weeks was at least as effective as doubling the dose (46). On balance, published data suggest that there is no increased risk of infections or other complications with increased doses or serum concentrations of anti-TNFs (47–53).

A number of studies based on small numbers of patients suggest that the addition of an immunomodulator can reverse ADAb formation and LOR, with some studies reporting that response could be regained in over half of all patients treated with anti-TNFs (54–59). The effects of immunomodulation can impact outcomes as early as 4 weeks after the addition of the immunomodulator (56), but on occasion they can take 2–3 months to achieve the full therapeutic effect. A course of steroids used as a bridge until the immunomodulator becomes effective may be an option for these patients.

### Switching Within Class to Another Anti-tumor Necrosis Factor

The use of TDM is helpful in the decision to switch within class to another anti-TNF. If the patient has developed ADAbs, and has previously benefited from an anti-TNF, then using another anti-TNF is a viable option as antibodies are specific for a given therapeutic molecule (a biosimilar is considered as the same anti-TNF in this specific context). This can be an effective alternative treatment strategy for patients with PNR or LOR if they have subtherapeutic drug concentrations and high levels of ADAbs. Based on published data and the authors’ clinical experience, Table 2 proposes levels of anti-TNFs that can be used to make clinical decisions at various clinical situations. Of note, some occurrences of PNR within 14 weeks from start of treatment may actually be a rapid LOR. Supplementary Table 1 provides a summary of selected relevant studies. Overall, available data suggest that switching within class to another anti-TNF following LOR is a viable strategy for a sub-group of patients and that TDM may help identify these patients (60). In addition, some small studies have reported clinical effectiveness following the use of a third, and even fourth, anti-TNF in some patients with CD following failure of two or more previous anti-TNFs (61–63). However, with the arrival of agents with alternative mechanisms of action, this option is not commonly used and may be reserved for certain patients with extra-intestinal manifestations (EIMs).

### Switching Out of Class to an Agent With a Different Mechanism of Action

If a patient does not achieve an adequate therapeutic response with an anti-TNF agent and has therapeutic or supratherapeutic drug levels (Table 3), then selecting an agent from a different treatment class is an appropriate approach. Treatments with alternative mechanisms of action, such as vedolizumab, ustekinumab, and tofacitinib (the latter has only been approved for UC), may be considered (Supplementary Table 2; 1–4, 12, 13).

**TABLE 3:** Potential factors affecting biologic drug levels/drug clearance (27, 173, 174).

Anti-drug antibody/drug complex formation

Concomitant treatment with immunomodulators
Leakage/loss to gut lumen
Inflammatory burden and drug consumption
CRP levels
TNF-α levels
FcRn (Brambell receptor) rescue system
Albumin levels
Body weight
Male gender

*CRP, C-reactive protein; FcRn, neonatal Fc receptor; TNF, tumor necrosis factor.*

The degree of efficacy following switching appears to vary by treatment type and previous therapy received. Singh et al. (64) reported that patients with PNR to anti-TNF agents were less likely to respond to second-line non-TNF biologics, as compared with patients who discontinued therapy due to intolerance. In addition, patients with PNR were less likely to respond to second-line ustekinumab than patients with LOR, but there was no difference between patients treated with vedolizumab. These findings may be attributed to the pharmacokinetics and pharmacodynamics of anti-TNFs in patients with PNR.

Some data suggest that biologic-naïve patients respond better to therapy than anti-TNF experienced patients. For example, *post-hoc* analyses of efficacy data from the GEMINI 2 and GEMINI 3 studies reported rates of response and remission to be numerically higher in patients with CD receiving vedolizumab as a first biologic than in patients who had previously experienced an inadequate response with anti-TNFs (65); clinical efficacy of vedolizumab appeared similar between the different types and number of anti-TNFs previously used. A meta-analysis based upon the CERTIFI and UNITI-1 clinical trials demonstrated that use of ustekinumab resulted in significantly higher responses than placebo in patients with LOR to anti-TNFs, those who had previously received ≥ 2 anti-TNFs, and in intolerant patients, but not in the case of PNR (66). Similar data have been published for patients with UC. A retrospective, observational cohort study of 722 patients with UC showed that vedolizumab-treated patients were more likely to achieve deep clinical remission than those treated with anti-TNFs and that this response was blunted by prior exposure to anti-TNFs (67). For ustekinumab, while an extensive literature review of clinical trials and real-world evidence noted that the efficacy of ustekinumab appears to be blunted by increased use of anti-TNF agents (68), an analysis of data from 95 UC patients from the ENEIDA registry found that number of previous biologic treatments did not affect the response to ustekinumab (69). Finally, exposure to anti-TNFs does not seem to affect the response to tofacitinib (70). Recently, ozanimod has been approved for the treatment of UC. Data from the phase III trial indicated that while treatment effect sizes for ozanimod were not different between anti-TNF naïve and experienced patients, rates of clinical response and clinical remission tended to favor the anti-TNF naïve group, mirroring what has been observed with vedolizumab and ustekinumab (71–73). Thus, while switching out of class can be an effective strategy for some patients, the reason for switching and the patient’s treatment history needs to be considered.

Prior immunogenicity to anti-TNFs does not appear to confer an increased risk of immunogenicity to ustekinumab or vedolizumab (74). The efficacy profiles of non-anti-TNF biologics may also influence treatment choice given that some may additionally treat EIMs of IBD. For example, while ustekinumab may be selected to treat UC or CD, it has also demonstrated efficacy in the treatment of paradoxical psoriasiform skin drug reactions and cutaneous manifestations of IBD (75).

It should also be borne in mind that PNR to anti-TNFs may be representative of a very sick patient who is thus less likely to respond to any biologic that is prescribed.

## Important Considerations for the Physician in Case of Non-response to Anti-tumor Necrosis Factors

Understanding different features that contribute to the efficacy of a certain drug may help to predict the therapeutic response in patients with IBD, thus providing the potential for personalized medicine (76, 77). Factors that are important to consider in this context are patient characteristics, comorbidities, disease phenotype, EIMs, the patient’s preferences, results from biomarker analyses, and treatment costs.

### Patient Characteristics

Patient-related factors, such as smoking and obesity, may increase the risk of LOR to anti-TNFs, suggesting the need for dose-escalation and alternative therapeutic approaches, such as possible lifestyle changes (34, 78, 79). Kennedy et al. (34) reported the need for dose intensification during induction for at-risk individuals (e.g., patients with obesity and regular smokers) and iterative dose adjustment to achieve target drug concentrations greater than those currently recommended had the potential to improve the durability and effectiveness of anti-TNF therapy in these patients.

The clinical effectiveness of anti-TNF therapy does not seem to differ between older and younger patients [≥60 vs. <60 years (80, 81)]. However, it has been reported that elderly patients had a higher risk of treatment failure with an initial anti-TNF agent compared with younger individuals (81, 82). Furthermore, the risk of serious adverse events and/or serious infections were significantly higher in those ≥60 years, which could be linked to potential comorbidities present (80).

For patients who are, or aim to become, pregnant, available guidelines suggest that all anti-TNFs are safe but could be discontinued at the start of the third trimester in patients with inactive disease (12). For patients with active disease or a high risk of relapse it is recommended to continue this treatment throughout pregnancy. Of note, the potential long-term effects of anti-TNFs on the unborn child throughout pregnancy are still unknown. However, as more data are being collected that are reassuring regarding long-term safety, experts in the field advocate an increasingly lower threshold for maintaining remission-protective treatment throughout pregnancy (83, 84). Although vedolizumab and ustekinumab are recommended to be used with caution, data to suggest that these agents are equally safe as anti-TNF agents are accumulating (85, 86). A recent analysis of 1,490 pregnancies among women with IBD across multiple centers in the US showed that biologic, thiopurine or combination therapy during pregnancy was not associated with increased maternal or fetal outcomes during the first year of life (85). Tofacitinib and ozanimod are thus far contraindicated in pregnancy; patients planning a pregnancy should not start either agent if alternative options are available. However, the data on tofacitinib are continually evolving and as such the decision to continue tofacitinib during pregnancy should be made in discussion with maternal-fetal medicine experts and following full explanation of uncertainty with the patient.

### Comorbidities

While the presence of comorbidities did not increase the risk of malignancies with anti-TNF use, the presence of cardiovascular disease was independently associated with the occurrence of serious infections (80) and no differences in the clinical effectiveness of anti-TNFs between patients with and without comorbidity with IBD were reported. Thus, patients with cardiovascular disease deemed to be at increased risk of infection may require additional assessment including an overview of the patient’s vaccination status prior to the use of anti-TNFs (see below). In patients with heart disease, such as congestive heart failure and rhythm disturbances, use of anti-TNFs may lead to worsening of cardiac function and alternative agents should be considered.

Patients with IBD may also develop serious infections due to the disease itself or its treatment, including biologic therapies. Increased susceptibility to infections with anti-TNFs, such as tuberculosis, prompts that physicians should try to detect and treat any latent infections and consider the overall risk of opportunistic infections prior to anti-TNF therapy (87, 88); of note, screening does not completely eliminate risk of infection. While the use of vaccinations is country dependent, guidance on opportunistic infections has recently been published by ECCO (89). All patient candidates for treatment with immunomodulators and/or targeted therapies or who are already receiving a targeted therapy should have their vaccine history checked and be provided with influenza and pneumococcal vaccines. While hepatitis B vaccination is usually performed in newborns, immunization status should be assessed and vaccination provided, where seronegative. Patients should be vaccinated for herpes zoster; while the old vaccine had to be administered at least 3 months prior to the initiation of anti-TNFs, the new inactivated vaccine, which is now readily available in many countries, can be given at any time and should therefore be recommended. Availability of the human papillomavirus vaccine varies by country, but should be used, where possible. The use of varicella vaccine should also be considered in those patients without any history of varicella (89). Vaccination for SARS-CoV-2 should also be recommended and this can be administered at any time (90). A recent report suggests that the vaccine response could be blunted by the use of anti-TNFs (91). However, other data suggest that IBD patients become seropositive after two doses of vaccine despite being under treatment with biologics (92) and that anti-TNFs could provide a protective effect against the disease (93). Taken together, booster doses are most likely beneficial for the patients with a blunted SARS-CoV-2 vaccine response, such as those under potent immunomodulatory/targeted therapy including IBD patients (94), and is recommended by local health authorities.

There are conflicting data on the safety of anti-TNFs in patients with active cancer or a history of cancer. In some patients the use of anti-TNFs may be an option in discussion with an experienced oncologist (95–97).

### Disease Phenotype

Some treatments may not be suitable for every CD or UC phenotype suggesting the need to select the management approach (e.g., biologics, immunomodulators, steroids and/or surgery) that best targets and addresses the structural complications of the specific patient (15, 98, 99). Importantly, multidisciplinary teams may be needed to support and implement appropriate therapeutic decisions (100).

Available guidelines recommend the use of infliximab for the induction and maintenance of remission in complex perianal fistulae in patients with CD (4, 12). Of note, fistula healing may be more likely in patients with higher infliximab trough levels, suggesting the need for personalized dosing in this setting (4). Adalimumab may also be used to manage complex perianal fistulae (4, 12). There is insufficient evidence regarding the effect of adding immunomodulators to anti-TNFs on fistula healing. In addition, there is currently insufficient evidence to recommend the use of vedolizumab for fistula healing in patients with CD (4, 12, 101). A recent meta-analysis including 198 patients from four studies demonstrated that use of vedolizumab led to the healing of perianal fistulas in approximately one third of patients (102). Finally, recent evidence suggests that ustekinumab may be effective against fistulas (103, 104).

For patients with acute severe UC, guidelines recommend the use of infliximab (1, 2), although no guidance is available regarding the routine use of intensive compared with standard infliximab dosing (1). There are indications that an accelerated dosing regimen could be beneficial (105, 106), however data are scarce and weak in this area thus far.

### Extra-Intestinal Manifestations

Up to 50% of patients with IBD experience EIMs (most commonly affecting the joints, skin, hepatobiliary tract, and eyes), which may parallel luminal disease activity or have an independent course (15, 107–109). For EIMs that are typically independent of intestinal disease activity choosing a more systemic therapy such as an anti-TNF, ustekinumab, or tofacitinib is preferred (15), although ustekinumab has not been shown to be effective in the management of axial arthropathies (110). In general, anti-TNFs appear to provide good response rates for cutaneous manifestations, arthritis, and ocular EIMs (100, 109). However, although data are sparse, ustekinumab may be preferred for some (but not all) cutaneous conditions, such as psoriasis or paradoxical psoriasiform drug reactions. Data remain both limited and conflicting for the use of vedolizumab for EIMs, with some suggesting an improvement in EIMs with treatment (111–113), while others suggest an increase in both the development and worsening of EIMs during treatment (114, 115).

### Patient Preference

Denesh et al. (116) recently reported that most patients with IBD prefer oral treatments. However, those patients who have already experienced biologic agents have a high level of acceptance for both subcutaneous and intravenous forms of medication (116). While oral formulations remain limited to the JAK inhibitors in IBD with regards to targeted therapies, subcutaneous and intravenous formulations of anti-TNFs, and subsequent anti-IL12/23s and integrin receptor antagonists allow additional patient choice which may support both patient empowerment and compliance (117–119). Of note, while many physicians think that patients prefer subcutaneous treatments over intravenous administration, this is not true for all patients (117). Some patients prefer IV administration with reasons given varying from less frequent dosing, convenience, the chance for interaction with hospital staff, and reassurance with medical presence (120).

### Biomarkers

Clinicians currently lack a valid tool that can predict an individual patient’s response to treatment and support both initial and subsequent therapeutic choices (76). Several candidate genetic, immunological, pharmacokinetic, and microbial biomarkers have been tested but due to low sensitivity and specificity, low practical feasibility and high costs associated with the suggested procedures, they are difficult to use in clinical practice. However, gene expression profiling, molecular imaging, and the microbiome have potential as future predictive factors of therapeutic efficacy (121).

Genetics may play a part in the therapeutic response given genetic risk alleles appear to predict PNR and durable response to anti-TNF therapy in patients with CD (122–124). A genome-wide association study by Sazonovs et al. reported a significant association between allelic variation in the HLA-DQA1 gene (HLA-DQA1*05 allele) and the development of ADAbs against anti-TNF agents. Thus, HLA-DQ1A*05 may serve as a useful biomarker of immunogenicity risk and testing for this variant might help physicians to decide whether they should receive anti-TNFs in combination with immunomodulator therapy (124). In addition, pharmacogenetic testing has the potential to support improved patient stratification, optimize treatment selection/dose, and to minimize harm caused by adverse drug reactions (125). Arijs et al. (126) reported a 100% accurate predictive gene signature for (non) response to infliximab in patients with Crohn’s colitis, although no such a predictive gene set could be identified for those with Crohn’s ileitis. Finally, Lee et al. (123) showed that the presence of a gene expression signature associated with CD8^+^ T cells was significantly associated with an increased risk of LOR in patients with CD.

The relationship between the gut microbiota and drugs used in the treatment of IBD may prove to be a source of future biomarkers (127). Aden et al. (128) suggest that metabolic network reconstruction and assessment of metabolic profiles of fecal samples could be used to identify patients with IBD likely to achieve clinical remission following anti-TNF therapy. Other studies suggest that low levels of *Faecalibacterium prausnitzii* and *Bacteroides* in the gut may predict relapse after discontinuation of anti-TNF therapy (129), and differences in gut microbiome may be able to differentiate between responders and non-responders (130–132).

While biomarkers predictive of efficacy constitute a promising area of research, their use is currently not recommended in clinical practice.

### Cost

Cost may also play a role in a physician’s choice of treatment in IBD (4), motivating the use of dose optimization or switching within class instead of switching out of class when no other factors influence treatment choice (Figure 2). Biologic drugs are associated with a high cost (133, 134) which may limit access and result in non-optimized initiation and duration of therapy (135). Due to the chronic nature of IBD and associated high clinical, economic and societal burden, an efficacious, yet cost-effective, approach to its long-term management needs to be considered (136–139). Clinical trials, analytical models and systematic reviews have consistently found TDM-guided strategies for the treatment of IBD to be cost-saving or cost-effective compared with standard treatment without TDM (140–144). The introduction of less costly biosimilar anti-TNF drugs has also been associated with significant cost reductions and has expanded access to biologics in countries, including low-income countries (145–147). The safety and effectiveness of biosimilars within IBD have been established in an increasing body of evidence since the introduction of the first infliximab biosimilar in 2013 (12, 148, 149). As such, anti-TNF biosimilars are strongly recommended as first-line therapy by regulatory authorities. The increasing availability of subcutaneous forms of biologics, such as infliximab (CT-P13), adalimumab, ustekinumab and vedolizumab, are also expected to affect cost considerations (150–152), and the relationship between cost and subcutaneous administration should be clarified.

## Conclusion

Several factors need to be considered when deciding upon the best treatment following PNR or LOR to anti-TNF therapy. Here we have presented evidence and experience-based decision-making factors that may help clinicians when deciding to switch within class or to switch out of class to a treatment with a different mechanism of action. Prior to switching treatment, it is critical to understand the reason as to why a patient is not responding, since this can affect management decisions and treatment choices. Switching within class should be considered in those patients with LOR due to high levels of ADAbs and/or where dose escalation has failed. The addition of an immunomodulator may also be considered, if ADAb-levels are low. Switching out of class appears to be an appropriate strategy in true PNR and those patients with a LOR with adequate serum trough drug levels. However, there is no consensus on the standardization of cut-off values for anti-TNF serum concentrations and some patients who are within a “therapeutic window” may still benefit from increased dosing. Treatment decisions also need to incorporate factors that may favor switching within, or out of, class including patient characteristics, disease phenotypes, comorbidities, EIMs, patient preference, and cost. Hopefully the guidance contained within this review will assist physicians in making informed treatment choices resulting in optimal long-term outcomes for their patients.

## Author Contributions

All authors made substantial contributions to the concept and design, analysis and interpretation of data, drafting of the manuscript or revising it critically for important intellectual content, and provided final approval of the manuscript.

## Conflict of Interest

MB-A has received financial support for traveling and educational activities from or has served as an advisory board member for Pfizer, MSD, Takeda, Abbvie, Kern, Janssen, Fresenius Kabi, Biogen, Ferring, Faes Farma, Shire Pharmaceuticals, Falk Pharma, Chiesi, Gebro Pharma, Otsuka Pharmaceuticals, and Tillotts Pharma. TB has received financial support for traveling and educational activities from or has served as an advisory board member for Takeda, Janssen, and Tillotts Pharma. IB has served as an advisory board member for Pfizer, MSD, Takeda, Abbvie, Galapagos, Amgen, Arena Pharma, BMS, Janssen, Fresenius Kabi, Biogen, Ferring, Dr. Falk Pharma, and Tillotts Pharma. MC has received lecture fees and has served as advisory board member for Takeda, Janssen, Shire, MSD, Abbvie, Ferring, Fresenius, and Biogen. JM has served as a speaker, consultant or advisory board member for AbbVie, Bayer, Biogen, Bristol-Myers Squibb, Ferring, Hospira, Janssen, MSD, Otsuka, Pfizer, Sandoz, Svar, Takeda, Tillotts, and UCB, and has received grant support from AbbVie, Ferring, Fresenius Kabi, Pfizer, and Takeda. SS has received personal fees from Janssen, Takeda, Galapagos, Celltrion, Falk Pharma, Tillots pharma, Cellgene, Pfizer, and Pharmacocosmos, and has received grant support from Takeda, Abbvie, Amgen, Tillots Pharm, and Biogen.

## Publisher’s Note

All claims expressed in this article are solely those of the authors and do not necessarily represent those of their affiliated organizations, or those of the publisher, the editors and the reviewers. Any product that may be evaluated in this article, or claim that may be made by its manufacturer, is not guaranteed or endorsed by the publisher.
