# Supplementary material for: Management of Non-response and Loss of Response to Anti-tumor Necrosis Factor Therapy in Inflammatory Bowel Disease
Source: Front Med (Lausanne). 2022 Jun 15;9:897936. doi: 10.3389/fmed.2022.897936 (PMC9241563; doi:10.3389/fmed.2022.897936)
Supplement: Supplementary file 1 [file Table_1.docx]

**Appendix**

**Table 1.** Results from selected IBD studies of anti-TNFs focusing on switching within class following primary non-response (PNR) or loss of response (LOR).

| Reference | Design | N/IBD | Definition of primary endpoint | Results |
| --- | --- | --- | --- | --- |
| Sandborn et al. 2004 | Open-label study  Infliximab→adalimumab  LOR or intolerance to infliximab | 24/CD | Clinical remission (CDAI score ≤150 points) and clinical response (decrease in CDAI ≥100 points) at 12 weeks post switching within class | - Switching within class from infliximab to adalimumab resulted in clinical remission and clinical response in 29% and 50% of patients, respectively, at Week 12 - Nineteen patients (79%) escalated their dose during weeks 4–6 - Adalimumab was well tolerated |
| Sandborn et al. 2007 | Randomised, double-blind, placebo-controlled study  Infliximab→adalimumab  Previous LOR or intolerance to infliximab | 301/CD | Induction of remission  at Week 4 | - 21% of patients (34 of 159) in the adalimumab group versus 7% (12 of 166) of those in the placebo group achieved remission Week 4 (p<0.001) - No patients receiving adalimumab vs 4/166 patients in the placebo group had a serious infection |
| Allez et al. 2010 | Treatment with adalimumab or certolizumab pegol as third anti-TNF  Retrospective study  LOR or intolerance with two previous anti-TNFs | 67/CD | Clinical response (decrease in HBI >3 points) at weeks 6 and 20 | - Clinical response observed in 41 (61%) at week 6 and 34 patients (51%) at week 20 |
| Gagniere et al. 2015 | Retrospective study  Infliximab→adalimumab→infliximab | 61/CD | Duration of infliximab treatment after reintroduction | - Median treatment duration after reintroduction was 16 months - Probability of remaining on infliximab was 60% and 51%, respectively, at 12 and 24 months - Remission was achieved in 42% of patients at weeks 6–8 after infliximab re-induction |
| Brandse et al. 2014 | Retrospective multicenter cohort study  Infliximab→adalimumab→infliximab | 29/CD | Sustained clinical benefit of infliximab retreatment (ongoing maintenance therapy with ‘clinical remission’ and/or ‘clinical response’ at all visits | - 18/29 (62%), patients were still on continued therapy of their second infliximab treatment at 18 months |
| Chaparro et al. 2012 | Historical cohort study in a community-based  gastroenterology practice  Adalimumab→infliximab | 15/CD | Luminal disease: complete response defined as HBI below or equal to ≤4 without steroids  Perianal CD: complete response defined as closure of all fistulas | - All patients who had interrupted adalimumab due to loss of efficacy regained response - All patients who discontinued adalimumab due to adverse events responded to infliximab and maintained response |
| Fumery et al. 2015 | Retrospective multicenter cohort study  Infliximab→adalimumab | 27/ Ped. CD | Adalimumab efficacy defined as  clinical remission (PGA=1) or clinical response (decrease of at  least 2 points of PGA score) 6 months after adalimumab initiation | - Adalimumab had clinical benefit as measured by the PGA score in 19 patients (70%) |
| Hinojosa et al. 2007 | Prospective, open-label, multicenter study | 50/CD | Luminal CD: clinical response (≥70-point reduction in CDAI score) and clinical remission (CDAI score <150)  Fistulizing CD: fistula remission (complete closure of all fistulas that were draining  at baseline) and fistula improvement (≥50%  decrease in the number of fistulas that were draining at baseline) | - Of 36 patients with luminal CD, 83% achieved clinical response and 42% achieved clinical remission at Week 4 - Of the 22 patients with fistulizing disease, 5 (23%) experienced fistula remission and 9 (41%) experienced fistula improvement at Week 4 |
| Ho et al. 2009 | Retrospective case-note review  study  Infliximab→adalimumab (89.8%) | 98/CD | Clinical remission (latest follow-up) defined by the regression of symptoms, in concordance with  both patient/physician global assessments (and normalization  of inflammatory markers) | - 60% of patients were in clinical remission at 1-year follow-up, with 30% and 55% requiring dose escalation to weekly therapy at 1-and 2-year follow-up, respectively - 17.8%, 42.2%, and 56.5% of patients failed to maintain clinical remission at 6-months, 1-year, and 2-year follow-up respectively following the initiation of adalimumab |
| Kassouri et al. 2020 | Retrospective multicenter study  Anti-TNF→vedolizumab or ustekinumab→anti-TNF | 100/CD | Treatment persistence, clinical response, and clinical remission at weeks 24 and 48 | - 13 patients were retreated with an anti-TNF, resulting in 13 (46.2%) clinical remission. Among the 29 patients included after ustekinumab failure, 12 were retreated with an anti-TNF, with 2 (16.7%) achieving clinical remission |
| Oussalah et al. 2009 | Retrospective single-center study  Adalimumab→infliximab | 53/CD | Maintenance of clinical  response defined as: (i) the absence of adverse  events leading to drug withdrawal; (ii) no major  abdominal surgery and (iii) no loss of clinical response  in initial responders | - Probability of maintaining clinical response was 77.2%, 67.8% and 50.8% at 26, 52, and 130 weeks respectively |
| Russi et al. 2017 | Retrospective case series | 8/CD | Review of patients’ medical history regarding demographics, disease characteristics, course of the disease, treatment history as well as current medication, and clinical data regarding efficacy of golimumab | - 3/8 patients showed a primary non-response. - Among 5 patients responding after induction, 1 patient showed a loss of response, and in 1 patient, treatment was ended due to side effect - Three patients have a continuous clinical response under golimumab |
| del Carmen R-Grau et al. 2016 | Retrospective, multicenter, open-label trial  Anti-TNF→ Anti-TNF | 118/CD | Response to anti-TNF was evaluated using the HBI at the end of the induction period for the short-term response and at the end of follow-up for the long-term response | - 51% of patients achieved remission in the short-term - The probability of remission was significantly lower in patients for whom the drug indication was perianal disease - The dose was increased in 33% of patients, and 37% achieved/regained remission - The probability of maintaining remission was 76%, 68%, and 64% at 12, 18 and 24 months, respectively |
| de Silva et al. 2012 | Retrospective single-center study  Second anti-TNF→third anti-TNF | 63/CD (57), UC (6) | Time to cessation of third anti TNF due to lack of response or adverse events | - Probability of remaining on the third anti-TNF was 0.69, 0.55, 0.37 and 0.25 at 6, 12, 24 and 36 months respectively |
| Peyrin-Biroulet et al. 2007 | Open-label study  Infliximab→adalimumab | 10/UC | Clinical improvement at Week 4 (defined as a decrease in CAI of >4) | - 4/10 patients (40%) benefited from subsequent adalimumab therapy; one patient achieved remission (CAI <4) and 3 had clinical improvement at Week 4 - 6 patients had no response (60%); 2/6 (33.3%) subsequently underwent colectomy |
| Taxonera et al. 2017a | Retrospective multicenter cohort study  Maintenance adalimumab (prior use of anti-TNFs: n=116) | 184/UC | Cumulative  probabilities of adalimumab failure-free survival and  colectomy-free survival | - 112 patients (60%) maintained corticosteroid-free clinical response - 69 patients (37%) had adalimumab failure - Anti-TNF-naïve patients had significantly lower adjusted rates of adalimumab failure (p<0.001), adalimumab dose escalation (p=0.002), and need for colectomy (p<0.004) - 76 patients (41%) needed dose escalation after secondary LOR; 47% of these regained response after escalation |
| Taxonera et al. 2017b | Retrospective multicenter cohort study  Golimumab used as first-, second-, and third-line anti-TNF | 140/UC | Duration of infliximab after introduction, discontinuation rate, clinical remission at week 6–8 (physician global assessment), dose escalation | - Response rates for golimumab were 75% as first anti-TNF, 70% as second anti-TNF (ns versus first anti-TNF), and 50% as third anti-TNF (p=0.007 vs first anti-TNF) - 22% of patients needed golimumab dose escalation: 71% of regained response after escalation |

CAI, clinical activity index; CD, Crohn’s disease; CDAI, Crohn's disease activity index; HBI, Harvey-Bradshaw index; LOR, loss-of-response; Ped, pediatric; PGA, Physician Global Assessment; TNF, tumor necrosis factor; UC, ulcerative colitis.

**Reference for Appendix Table 1**

Sandborn WJ, Hanauer S, Loftus EV, Jr., Tremaine WJ, Kane S, Cohen R, et al. An Open-Label Study of the Human Anti-Tnf Monoclonal Antibody Adalimumab in Subjects with Prior Loss of Response or Intolerance to Infliximab for Crohn's Disease. Am J Gastroenterol (2004) 99(10):1984-9. Epub 2004/09/28. doi: 10.1111/j.1572-0241.2004.40462.x..

Sandborn WJ, Rutgeerts P, Enns R, Hanauer SB, Colombel JF, Panaccione R, et al. Adalimumab Induction Therapy for Crohn Disease Previously Treated with Infliximab: A Randomized Trial. Ann Intern Med (2007) 146(12):829-38. Epub 2007/05/02. doi: 10.7326/0003-4819-146-12-200706190-00159.

Allez M, Vermeire S, Mozziconacci N, Michetti P, Laharie D, Louis E, et al. The Efficacy and Safety of a Third Anti-Tnf Monoclonal Antibody in Crohn's Disease after Failure of Two Other Anti-Tnf Antibodies. Aliment Pharmacol Ther (2010) 31(1):92-101. Epub 2009/08/28. doi: 10.1111/j.1365-2036.2009.04130.x.

Gagniere C, Beaugerie L, Pariente B, Seksik P, Amiot A, Abitbol V, et al. Benefit of Infliximab Reintroduction after Successive Failure of Infliximab and Adalimumab in Crohn's Disease. J Crohns Colitis (2015) 9(4):349-55. Epub 2014/12/31. doi: 10.1093/ecco-jcc/jju024.

Brandse JF, Peters CP, Gecse KB, Eshuis EJ, Jansen JM, Tuynman HA, et al. Effects of Infliximab Retreatment after Consecutive Discontinuation of Infliximab and Adalimumab in Refractory Crohn's Disease. Inflamm Bowel Dis (2014) 20(2):251-8. Epub 2014/01/01. doi: 10.1097/01.MIB.0000438248.14218.1d.

Chaparro M, Andreu M, Barreiro-de Acosta M, Garcia-Planella E, Ricart E, Domenech E, et al. Effectiveness of Infliximab after Adalimumab Failure in Crohn's Disease. World J Gastroenterol (2012) 18(37):5219-24. Epub 2012/10/16. doi: 10.3748/wjg.v18.i37.5219.

Fumery M, Jacob A, Sarter H, Michaud L, Spyckerelle C, Mouterde O, et al. Efficacy and Safety of Adalimumab after Infliximab Failure in Pediatric Crohn Disease. J Pediatr Gastroenterol Nutr (2015) 60(6):744-8. Epub 2015/05/23. doi: 10.1097/MPG.0000000000000713..

Hinojosa J, Gomollon F, Garcia S, Bastida G, Cabriada JL, Saro C, et al. Efficacy and Safety of Short-Term Adalimumab Treatment in Patients with Active Crohn's Disease Who Lost Response or Showed Intolerance to Infliximab: A Prospective, Open-Label, Multicentre Trial. Aliment Pharmacol Ther (2007) 25(4):409-18. Epub 2007/02/03. doi: 10.1111/j.1365-2036.2006.03232.x.

Ho GT, Mowat A, Potts L, Cahill A, Mowat C, Lees CW, et al. Efficacy and Complications of Adalimumab Treatment for Medically-Refractory Crohn's Disease: Analysis of Nationwide Experience in Scotland (2004-2008). Aliment Pharmacol Ther (2009) 29(5):527-34. Epub 2009/02/03. doi: 10.1111/j.1365-2036.2008.03919.x.

Kassouri L, Amiot A, Kirchgesner J, Treton X, Allez M, Bouhnik Y, et al. The Outcome of Crohn's Disease Patients Refractory to Anti-Tnf and Either Vedolizumab or Ustekinumab. Dig Liver Dis (2020) 52(10):1148-55. Epub 2020/08/24. doi: 10.1016/j.dld.2020.07.031.

Oussalah A, Babouri A, Chevaux JB, Stancu L, Trouilloud I, Bensenane M, et al. Adalimumab for Crohn's Disease with Intolerance or Lost Response to Infliximab: A 3-Year Single-Centre Experience. Aliment Pharmacol Ther (2009) 29(4):416-23. Epub 2008/11/28. doi: 10.1111/j.1365-2036.2008.03902.x.

Russi L, Scharl M, Rogler G, Biedermann L. The Efficacy and Safety of Golimumab as Third- or Fourth-Line Anti-Tnf Therapy in Patients with Refractory Crohn's Disease: A Case Series. Inflamm Intest Dis (2017) 2(2):131-8. Epub 2018/07/19. doi: 10.1159/000481400.

del Carmen R-Grau M, Chaparro M, Mesonero F, Barreiro-de Acosta M, Castro L, Castro M, et al. Effectiveness of Anti-Tnfalpha Drugs in Patients with Crohn's Disease Who Do Not Achieve Remission with Their First Anti-Tnfalpha Agent. Dig Liver Dis (2016) 48(6):613-9. Epub 2016/03/20. doi: 10.1016/j.dld.2016.02.012..

de Silva PS, Nguyen DD, Sauk J, Korzenik J, Yajnik V, Ananthakrishnan AN. Long-Term Outcome of a Third Anti-Tnf Monoclonal Antibody after the Failure of Two Prior Anti-Tnfs in Inflammatory Bowel Disease. Aliment Pharmacol Ther (2012) 36(5):459-66. Epub 2012/07/13. doi: 10.1111/j.1365-2036.2012.05214.x.

Peyrin-Biroulet L, Laclotte C, Roblin X, Bigard MA. Adalimumab Induction Therapy for Ulcerative Colitis with Intolerance or Lost Response to Infliximab: An Open-Label Study. World J Gastroenterol (2007) 13(16):2328-32. Epub 2007/05/19. doi: 10.3748/wjg.v13.i16.2328.

Taxonera C, Iglesias E, Munoz F, Calvo M, Barreiro-de Acosta M, Busquets D, et al. Adalimumab Maintenance Treatment in Ulcerative Colitis: Outcomes by Prior Anti-Tnf Use and Efficacy of Dose Escalation. Dig Dis Sci (2017) 62(2):481-90. Epub 2016/12/21. doi: 10.1007/s10620-016-4398-5.

Taxonera C, Rodriguez C, Bertoletti F, Menchen L, Arribas J, Sierra M, et al. Clinical Outcomes of Golimumab as First, Second or Third Anti-Tnf Agent in Patients with Moderate-to-Severe Ulcerative Colitis. Inflamm Bowel Dis (2017) 23(8):1394-402. Epub 2017/07/04. doi: 10.1097/MIB.0000000000001144.

**Table 2.** Results from selected IBD studies focusing on switching out of class from anti-TNFs to a biologic with a different mechanism of action.

| Reference | Design | N/IBD | Definition of primary endpoint | Results |
| --- | --- | --- | --- | --- |
| Biemans et al. 2020 | Registry  Anti-TNFs→vedolizumab (n=128) or ustekinumab (n=85) | 213/CD | Proportion of patients  in corticosteroid-free clinical remission (HBI ≤4) at Week 52 | - Ustekinumab-treated patients were more likely to achieve corticosteroid-free clinical remission (OR 2.58; 95% CI: 1.36, 4.90; p=0.004) |
| Liefferinckx et al. 2019 | Observational, national, retrospective multicenter study  ≥1 anti-TNF (69.7% were exposed to two anti-TNFs and vedolizumab)→ustekinumab | 152/CD | Clinical response (reduction in the HBI of ≥3) and clinical remission (HBI of ≤4) at 12 months | - After 1 year, 42.1% and 25.7% of patients had experienced clinical response and clinical remission, respectively |
| Sands et al. 2017 | Post hoc analyses of two randomized, placebo-controlled studies  Anti-TNF failure | 960/CD | Induction treatment endpoints  included clinical remission (CDAI score of ≤150) and enhanced  clinical response (≥100-point decrease from baseline in the CDAI score [CDAI-100 response]) at Week 6 and Week 10 | - Among patients who responded to vedolizumab induction at Week 6, 27.7% of patients were in remission with vedolizumab at Week 52 (versus 12.8% with placebo) |
| Townsend et al. 2020 | Randomised, controlled multicenter study  Anti-TNF→ vedolizumab (n=85)  Anti-TNF→ ustekinumab (n=45) | 130/CD | Difference in steroid-free remission rates at end of induction (2 months) and at 12 months | - Steroid-free remission was higher among ustekinumab-treated patients at 2 months (p=0.038) and 12 months (p=0.095) - More patients treated with ustekinumab remained on therapy at the end of 12 months (84.4% vs 61.5%, p=0.007) |
| Mader et al. 2020 | Real-life observational study  Anti-TNF→vedolizumab | 101/UC  117/CD | Remission defined as calprotectin <200 mg/kg stool and/or mucosal healing determined by endoscopy between Months 4 and 8 (T1) and Months 12 and 16 (T2) after vedolizumab induction | - Remission was reported in 50.5% (110/218) of patients in T1 (48.7% CD and 52.5% UC) and 46.8% (102/218) in T2 (47% CD and 46.5% UC) - A significantly higher remission rate was achieved in T2 among anti-TNF naïve patients (57.7%) compared with anti-TNF-experienced patients with UC (34.7%; p=0.02). In patients with CD, no difference was reported for either evaluation interval. |
| Weisshof et al. 2019 | Retrospective observational study  Anti-TNF→tofacitinib  93% of the patients previously failed treatment with anti-TNFs | 53/UC  4/CD  1/pouchitis | Response to treatment was determined as defined by the patient’s provider and the decision to continue therapy. Response was defined as symptomatic improvement but not resolution, and remission was defined as complete resolution of clinical symptoms | - At 8 weeks of treatment, 21 (36%) patients achieved a clinical response and 19 (33%) achieved clinical remission - Steroid-free remission at 8 weeks was achieved in 15 (26%) patients - Of 48 patients followed for 26 weeks, 21% had clinical, steroid-free remission - Of 26 patients followed for 12 months, 27% were in clinical, steroid-free remission |
| Sands et al. 2019 | Randomized, double-  blind, double-dummy, active-controlled superiority  study  Anti-TNF (not adalimumab)→ vedolizumab (n=81)  Anti-TNF (not adalimumab)→ adalimumab (n=79) | 160/UC | Clinical remission at Week 52 (defined as a total score of ≤2 on the Mayo scale and no subscore >1 on any of the four components) | - Clinical remission at Week 52 was observed in 20.3% of the vedolizumab group and 16.0% of the adalimumab group |
| Hupé et al. 2020 | Retrospective multicenter observational study  Adalimumab or golimumab→infliximab (n=154) or vedolizumab (n=71) | 225/UC | Clinical remission (partial Mayo score ≤1) at Week 14 | - Clinical remission at Week 14 was achieved in 40/154 (26%) patients treated with infliximab and in 35/71 (49%) treated with vedolizumab   (p=0.001)   - After a propensity score matching analysis, this difference remained significant (p=0.02) |
| Lair-Mehiri et al. 2020 | National French cohort study  1≥ anti-TNF agent→vedolizumab→tofacitinib  31 (81.6%) patients were primary non-responders to an anti-TNF | 38/UC | Clinical remission was defined as a partial Mayo score <3 with a combined stool frequency and rectal bleeding subscore ≤1 | - Steroid-free clinical remission was observed in 13 (34%) patients at Week 48 |

CD, Crohn’s disease; CI, confidence interval; HBI, Harvey Bradshaw Index; TNF, tumour necrosis factor; UC, ulcerative colitis.

**References for Appendix Table 2**

Biemans VBC, van der Woude CJ, Dijkstra G, van der Meulen-de Jong AE, Lowenberg M, de Boer NK, et al. Ustekinumab Is Associated with Superior Effectiveness Outcomes Compared to Vedolizumab in Crohn's Disease Patients with Prior Failure to Anti-Tnf Treatment. Aliment Pharmacol Ther (2020) 52(1):123-34. Epub 2020/05/23. doi: 10.1111/apt.15745.

Liefferinckx C, Verstockt B, Gils A, Noman M, Van Kemseke C, Macken E, et al. Long-Term Clinical Effectiveness of Ustekinumab in Patients with Crohn's Disease Who Failed Biologic Therapies: A National Cohort Study. J Crohns Colitis (2019) 13(11):1401-9. Epub 2019/04/17. doi: 10.1093/ecco-jcc/jjz080.

Sands BE, Sandborn WJ, Van Assche G, Lukas M, Xu J, James A, et al. Vedolizumab as Induction and Maintenance Therapy for Crohn's Disease in Patients Naive to or Who Have Failed Tumor Necrosis Factor Antagonist Therapy. Inflamm Bowel Dis (2017) 23(1):97-106. Epub 2016/12/09. doi: 10.1097/MIB.0000000000000979.

Townsend T, Razanskaite V, Dodd S, Storey D, Michail S, Morgan J, et al. Comparative Effectiveness of Ustekinumab or Vedolizumab after One Year in 130 Patients with Anti-Tnf-Refractory Crohn's Disease. Aliment Pharmacol Ther (2020) 52(8):1341-52. Epub 2020/09/22. doi: 10.1111/apt.16057.

Mader O, Juillerat P, Biedermann L, Michetti P, Hruz P, Pittet V, et al. Factors Influencing the Outcome of Vedolizumab Treatment: Real-Life Data with Objective Outcome Measurements. United European Gastroenterol J (2021) 9(3):398-406. Epub 2020/11/19. doi: 10.1177/2050640620965106.

Weisshof R, Aharoni Golan M, Sossenheimer PH, El Jurdi K, Ollech JE, Pekow J, et al. Real-World Experience with Tofacitinib in Ibd at a Tertiary Center. Dig Dis Sci (2019) 64(7):1945-51. Epub 2019/02/09. doi: 10.1007/s10620-019-05492-y.

Sands BE, Peyrin-Biroulet L, Loftus EV, Jr., Danese S, Colombel JF, Toruner M, et al. Vedolizumab Versus Adalimumab for Moderate-to-Severe Ulcerative Colitis. N Engl J Med (2019) 381(13):1215-26. Epub 2019/09/26. doi: 10.1056/NEJMoa1905725.

Hupé M, Riviere P, Nancey S, Roblin X, Altwegg R, Filippi J, et al. Comparative Efficacy and Safety of Vedolizumab and Infliximab in Ulcerative Colitis after Failure of a First Subcutaneous Anti-Tnf Agent: A Multicentre Cohort Study. Aliment Pharmacol Ther (2020) 51(9):852-60. Epub 2020/03/24. doi: 10.1111/apt.15680.

Lair-Mehiri L, Stefanescu C, Vaysse T, Laharie D, Roblin X, Rosa I, et al. Real-World Evidence of Tofacitinib Effectiveness and Safety in Patients with Refractory Ulcerative Colitis. Dig Liver Dis (2020) 52(3):268-73. Epub 2019/11/17. doi: 10.1016/j.dld.2019.10.003.
